# Seizure detection with reduced electroencephalogram channels: research trends and outlook

**DOI:** 10.1098/rsos.230022

**Published:** 2023-05-03

**Authors:** Christina Maher, Yikai Yang, Nhan Duy Truong, Chenyu Wang, Armin Nikpour, Omid Kavehei

**Affiliations:** ^1^ School of Biomedical Engineering, Faculty of Engineering, The University of Sydney, Sydney, New South Wales 2006, Australia; ^2^ Brain and Mind Centre, The University of Sydney, Sydney, New South Wales 2006, Australia; ^3^ Translational Research Collective, Faculty of Medicine and Health, The University of Sydney, Sydney, New South Wales 2050, Australia; ^4^ Sydney Neuroimaging Analysis Centre, Camperdown, New South Wales 2050, Australia; ^5^ Central Clinical School, Faculty of Medicine and Health, The University of Sydney, Sydney, New South Wales 2006, Australia; ^6^ Translational Research Collective, Faculty of Medicine and Health, The University of Sydney, Camperdown, New South Wales 2050, Australia

**Keywords:** electrode, electroencephalogram, epilepsy, seizure detection, machine learning, patient care

## Abstract

Epilepsy is a prevalent condition characterized by recurrent, unpredictable seizures. Monitoring with surface electroencephalography (EEG) is the gold standard for diagnosing epilepsy, but a time-consuming, uncomfortable and sometimes ineffective process for patients. Further, using EEG over a brief monitoring period has variable success, dependent on patient tolerance and seizure frequency. The availability of hospital resources and hardware and software specifications inherently restrict the options for comfortable, long-term data collection, resulting in limited data for training machine-learning models. This mini-review examines the current patient journey, providing an overview of the current state of EEG monitoring with reduced electrodes and automated channel reduction methods. Opportunities for improving data reliability through multi-modal data fusion are suggested. We assert the need for further research in electrode reduction to advance brain monitoring solutions towards portable, reliable devices that simultaneously offer patient comfort, perform ultra-long-term monitoring and expedite the diagnosis process.

## 1. Introduction

Epilepsy is a severe neurological condition that affects millions of people worldwide. The main symptoms are recurrent seizures which can be a traumatic experience for the individual [1]. Epilepsy diagnosis requires attendance at a specialized epilepsy clinic or hospital. The incidence of false-positive diagnosis reportedly ranges from 2% to 71% [2]. Hospital-based monitoring depletes financial, mental, physical and time resources. In 2016, the global prevalence of active epilepsy was 45.9 million individuals with 126 055 epilepsy-related deaths, 13.5 million disability-adjusted-life-years, 5.9 million years of life lost (YLL) and 7.5 million years of life with a disability (YLD) [3]. In low- and middle-income countries, greater premature mortality has been associated with lack of access to medical facilities [4]. These statistics emphasize the need for improved tools for epilepsy diagnosis and treatment.

Surface or scalp electroencephalography (herein termed ‘EEG’), recorded from the head surface rather than inside the scalp, has been a requisite initial diagnostic test in epilepsy since the 1940s [5]. Clinically, the fundamental role of scalp EEG is to non-invasively capture electrical signals that can indicate states of seizure (‘ictal’) or non-seizure (‘interictal’), in turn enabling identification of the seizure onset zone. For individuals who require intracranial monitoring, scalp EEG is integral in assessing the optimal location for electrode placement. Likewise, in the assessment of surgical candidacy or other minimally invasive treatments, such as stereotactic laser thermal ablation and responsive neurostimulation (RNS), scalp EEG can guide the initial delineation of the seizure onset zone. The likelihood of surgical success can also be determined using scalp EEG [6–8].

This mini-review focuses on surface EEG rather than alternative EEG monitoring such as intracranial or stereotactic EEG (sEEG). Briefly, sEEG involves the placement of electrodes in the deep brain structures, where the electrical activity cannot be effectively monitored from the head surface. A more extensive explanation of intracranial, microelectrode-based recordings in epilepsy is made elsewhere [9]. This review will first introduce the concepts of the electrode, channel and the current seizure logging and detection process, where the number of channels is most important. Next, channel selection methods and the scenarios where they may be most applicable are presented. Lastly, the opportunities for channel reduction are discussed, including options to augment EEG data with multi-modal physiological data and the future of EEG wearables.

## 2. Electrode versus channel—what is the difference?

In scalp EEG, electrodes serve as a crucial hardware component for recording electrical activity in the brain [10]. The conventional EEG electrode, known as a ‘wet electrode’, is a disc-shaped silver/silver chloride sensor. The sensor is coated with conductive gel, securing the electrode to the scalp and enhancing the electrical signal quality. Scalp EEG electrodes are typically classified as ‘macro-electrodes’ with a diameter greater than 3 mm, while ‘micro-electrodes’ used in sEEG have a diameter of less than 1 mm. When two electrodes capture a signal between them, they are considered a ‘channel’. The amplifier, which doubles as the recording device, is connected to the electrode via wires. The user selects the channels in most cases since EEG software typically enables channel manipulation through the ‘montage’ settings. Reducing the number of physical electrodes on the scalp could decrease the number of available channels, limiting the number of channels available during the EEG analysis phase. Consequently, the question arises regarding the impact of reducing the physical electrodes used during recording.

## 3. How is electroencephalography recorded and what does it represent?

The electrical activity recorded from the scalp generally represents the summation of the postsynaptic potentials from the electrical dipoles between the soma and apical dendrites of large populations of pyramidal neurons. Therefore, EEG recordings continuously monitor neural activity and capture spontaneous or induced changes. Visually, scalp EEG enables the analysis of the spectral characteristics of the electrical signals, usually ranging in frequency from 1 to 30 Hz. Also captured in the spectral frequencies are evoked potentials—electrical signals that vary due to external or internal stimuli. Changes in EEG signals could represent spontaneous electrical brain activities that exhibit dynamic, stochastic, nonlinear, non-stationary and complex behaviour with high temporal resolution. Given the electrode position on the scalp, the spectral power can be used as a marker of local synchronization. Such markers can then be altered with computations to infer synchronization between two distant regions in the brain.

## 4. The current patient journey

**Figure 1.**
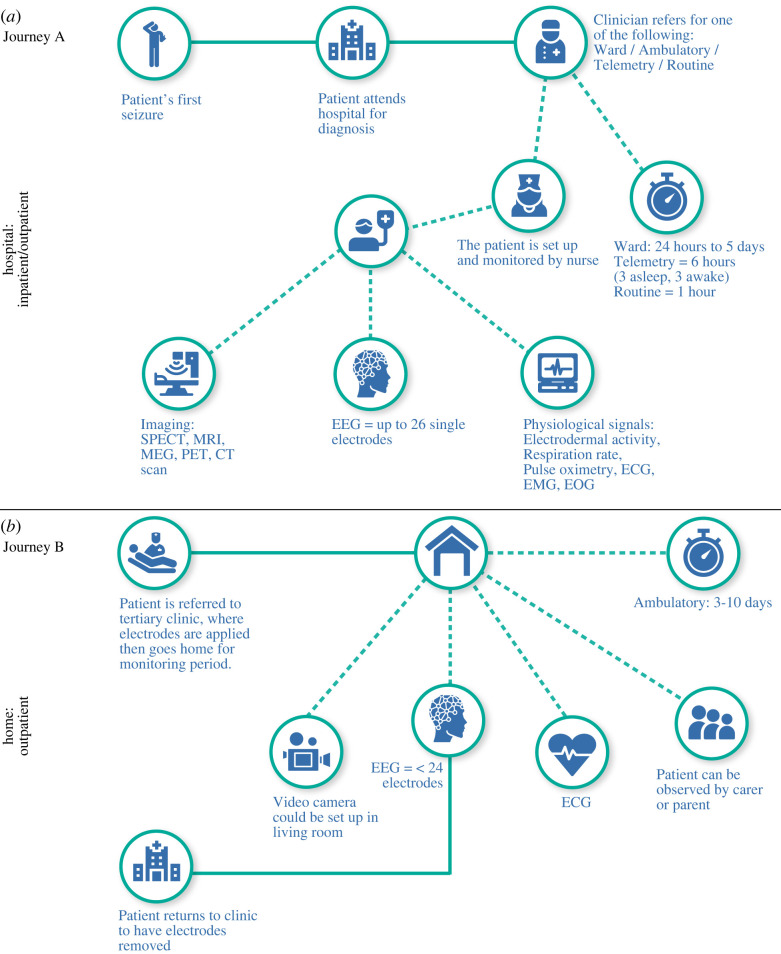
Schematic of the typical inpatient and outpatient journey. In the hospital setting (*a*, Journey A), a carer accompanies the patient for the entire ward stay. Patient comfort, mobility and limited hospital resources restrict the time a patient can spend in the hospital and whether they can even be accommodated. Conversely, the home setting (*b*, Journey B) facilitates patient comfort and mobility. Patients can continue their daily activities while being monitored for an extended duration. Unfortunately, improving patient comfort and mobility through home monitoring may come at a potentially higher cost financially than a ward stay. Further, electrodes do not last long on the scalp; the patient must inevitably return to the hospital or pay for a home visit, where a technician can perform electrode adjustment, replacement or removal. Using an in-home video camera during the monitoring period increases the potential for, and risk of, data privacy issues and data breaches. Improvements to this imperfect process lie in extending the research in electrode reduction to enable long-term wearable solutions for the patient. Key: SPECT: single photon emission computed tomography, MRI: magnetic resonance imaging, MEG: magnetoencephalography, PET: positron emission tomography, CT: computerized tomography, ECG: electrocardiogram, EMG: electromyography, EOG: electrooculogram.

The lengthy epilepsy diagnosis process involves multiple tests and continuous monitoring of EEG and video (vEEG). The diagnostic process can occur in a hospital ward or via an ‘ambulatory’ service where the EEG device is worn at home. Figure 1 depicts the journey through the healthcare system from the first seizure to epilepsy diagnosis. The various monitoring scenarios (ward/ambulatory/routine) begin when a patient is referred to a tertiary epilepsy centre after a first seizure. A full-time carer must be present if ward monitoring is required, placing additional strain on carers and the patient's ecosystem. If a ward stay is not required or not possible due to lack of resources, a clinician may obtain a 20–40 min routine EEG recording (as is the case in Germany).

The possibility of delayed diagnosis or misdiagnosis arises through several aspects of the diagnosis process. First, if seizures are not evident on the EEG or vEEG, there is a high likelihood of inconclusive EEG results, where epilepsy is neither confirmed nor excluded. Thus the risk of misdiagnosis must be weighed against that of a false positive diagnosis, a complex decision for clinicians with severe outcomes for patients [2]. The nature of current scalp electrodes presents complications regardless of the monitoring setting. Existing devices require abrasive conductive gel for 19 or more electrodes, which can irritate or even damage the skin. The water-based conductive gel dries over time, impacting the signal impedance. Individual characteristics, such as hair and scalp thickness and pathological state, can influence EEG recordings. Electrode application for individuals with neurological or sensory disorders is especially challenging, and the electrodes can fall off easily during a motor seizure. This requires electrodes to be re-applied multiple times in a monitoring period, resulting in technician variance.

A valuable extension of the relatively short EEG monitoring periods could be sending the individual home with a wearable device suitable for long-term daily EEG recordings, which a reduced electrode set would make feasible. Yet this path has its challenges. From an engineering perspective, technological advances have made automated seizure detection and device portability a reality. The longest scalp EEG recording using wet or dry electrodes is approximately 10 days. From a patient's perspective, mobile EEG systems must be comfortable and appealing to wear, and current designs are lacking. Since patients' self-record of their seizures can be inaccurate, achieving a balance between technical features and patient comfort in a portable EEG system would further evolve home EEG monitoring.

## 5. Seizure logging and detection—current state

### 5.1. Human-based manual seizure detection

Currently, EEG is the gold standard to detect interictal, ictal and subclinical epileptic activity, including critical conditions like status epilepticus [11,12]. Typical EEG monitoring requires up to 19 head and two reference electrodes, based on the international 10–20 electrode placement system [13]. This standard electrode placement applies in various settings, including epilepsy centres and hospital inpatients. In ambulatory monitoring in the home setting, a device with up to 19 electrodes is often used.

**Figure 2.**
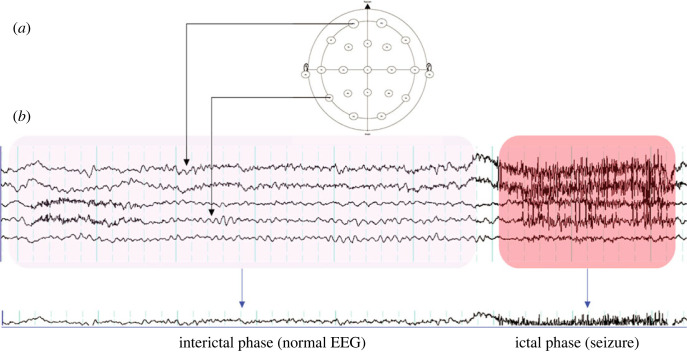
EEG signals are recorded from electrodes placed on the scalp in the 10–20 format (*a*). The signals are then displayed on readout software. An EEG technician, neurologist or epileptologist then visually identifies and labels the interictal, pre-ictal and ictal phases (*b*).

Electrode positioning and a typical ictal event are illustrated in figure 2. Here, an interictal period precedes an ictal event (red highlighted section), shown on a montage of five channels out of the standard 21. Recording EEG data with a complete electrode set over an extended period yields reliable, interpretable EEG that can inform clinical decisions. In routine clinical practice, human experts (experts) may be highly trained nurses, EEG technicians, neurophysiologists and neurologists who identify, label, and classify the seizure onset and type. The experts label the EEG with the seizure time, length and location. However, this manual process is highly time-consuming and can be error-prone.

### 5.2. Computer-based automated seizure detection

Computer-automated techniques, such as machine learning (ML) algorithms, can detect and label seizures. Channel selection involves reducing the number of electrodes to be analysed or utilizing a range of feature selection and classification techniques, or both [14–18]. The model is trained on individual patients or across all patients, then tested on the dataset of interest [19,20]. The quantity of recorded EEG signals often plays a fundamental role in the success of the seizure detection model [21], as does the seizure type [22].

In automated seizure detection, digital signal processing allows extracting typical EEG signal features, which can be raw or engineered. Prior human studies examined engineered features such as relative average amplitude, relative scale energy, average cross-correlation function, relative power, bounded variation [23], phase-amplitude coupling [24], root mean square [25], energy [26], median frequency [27], entropy [28,29], correlation dimensions [30–32], maximal Lyapunov exponent [33], skewness and kurtosis [34] and even models from game theory [35]. Such features may be the sole focus of the model [28] or combined and applied to a selected EEG frequency band [36–38]. Raw features can be directly extracted from the raw EEG or after signal processing with short-time Fourier transform (STFT) or wavelet transform.

Conventional ML methods can then be trained to classify the EEG signals based on the selected EEG features [38–40]. During training, the EEG features are fed to the model, and additional threshold mechanisms may be applied to the output via the selected ‘classifier’ technique [41]. Standard classifier techniques include but are not limited to Bayesian, K-nearest neighbours (KNN), decision tree, random forest and support vector machine (SVM) classifiers [21]. Such classification models typically group patients based on their seizure type. EEG-based biomarkers can represent features such as seizure susceptibility [42] and intra-patient seizure morphology variability [43], which may improve automated seizure detection models. However, convolutional neural networks (CNN) are advantageous over conventional ML models as they allow training and inference directly from the raw data, spawning many encouraging studies from the seizure detection community [44–47]. Indeed, our recent work used a Bayesian CNN-based model to show that interictal slowing activity is a promising feature for seizure susceptibility prediction [42].

The reliance of automated seizure detection models on engineered features highlights their vulnerability to changes in the brain state, electrode count, recording region, EEG artefacts and signal noise [48,49]. Subsequently, the reliability of channel selection algorithms is also affected when models are fed insufficient data. Thus it is evident why electrode reduction is considered problematic and receives little enthusiasm in the clinical setting. However, channel selection algorithms are improving; several of the current channel selection models and their viability will now be discussed.

### 5.3. Channel selection methods

Channel selection involves applying a computational or statistical methodology such as machine learning to extract the channels with the most importance at any given moment. If machine learning techniques are used, then sensitivity and specificity scores are applied to the result, indicating whether the model successfully extracted the most important channels. Sensitivity is taken to measure the true positive rate (TPR), whereas specificity measures the true negative rate (TNR). Sensitivity and specificity act as widely accepted, essential measures to evaluate the machine learning model's performance. The important channels can be selected across a group of patients, or they can be patient specific [50], inferring unique channels for individual patients depending on their epilepsy type and onset zone [51,52].

A foundational study by Shih *et al.* used the so-called ‘greedy backward elimination’ algorithm to select a subset of seven features that produce the lowest false positive rate from each channel [53]. The reduced number of channels was between 18 and 4.6, achieving improvement in false-positive rate (FPR), from 0.35 to 0.19 h^−1^, yet sensitivity and detection delay worsened, from 99% to 97% and 7.8 s to 11.2 s, respectively. More recently, Moctezuma and Molinas compared the popular greedy backward elimination algorithm with two versions of the non-dominated sorting genetic algorithm (NSGA), NSGA-II and NSGA-III [54]. They achieved an accuracy between 0.98 and 1 using one to two channels, comparable to their detection result with the full electrode count (accuracy ranged from 1 to 0.97).

The role of variance contributed by individual channels has also been widely explored. Duun-Henriksen *et al.* reduced channels by selecting the largest variance in channels, achieving a detection performance on three channels near equivalent to a clinical neuro-physiologist's review on the same dataset EEG [55]. Birjandtalab *et al.* used a random forest algorithm to determine which channels contributed the most variation to discrimination of seizure versus non-seizure events [56]. However, their minimal channel reduction, from 23 to 18 channels, provides little difference in a clinical application where 19 electrodes are standard. Bhattacharya and colleagues pre-selected 5 out of 23 channels to perform a multivariate analysis of EEG signals [57]. The one channel that displayed the least standard deviation informed the selection of the remaining four channels based on their interdependency level and similarity to the first channel. The model's performance ranged from 0.95 to 0.99, making it comparable to other channel selection methods.

A unique approach by Shah *et al.* focused on domain knowledge to inform the channel selection [58]. They exploited insights on brain hemisphere function, the proximity of a given electrode to other electrodes, electrode position on the scalp, and the region the electrode covered in terms of signal capture. Their eight-electrode montage produced the most favourable results (sensitivity 30.66%, specificity 88.79%) yet their work affirmed the scarcity of superior techniques that permit electrode reduction while maintaining model sensitivity. A prospective study by Kjaer *et al.* investigated automated seizure detection in a paediatric population of six patients aged 7–12 years [16]. Using three electrodes with references, they achieved a mean sensitivity of 98.4%, a specificity of 100% and a mean false detection rate of 5.5 per 24 h. Their study lacked comparisons with a full electrode set, but the prospective design and facilitation of at-home device usage were advantageous. The reviewed studies indicate that the availability of sufficient and reliable EEG data is critical to translating automated seizure detection into the clinical realm.

## 6. Limitations of reduced electrodes/channels

Despite the clear benefits of using fewer electrodes, the recording of less data, or poor spatial resolution compromising the quality and reliability of the diagnostic method remains a concern. Thus, reducing electrodes requires a trade-off between electrode count and signal quantity and quality.

The impact of electrode reduction on human and computer seizure detection performance is problematic. Deterioration in performance is attributed to the reduced availability and accuracy of the EEG data and technological challenges prevail in data collection and hardware design. Stevenson *et al.* [59] found electrode reduction negatively impacted visual interpretation by human experts. They misjudged seizure burden, and seizure annotation was significantly higher when they used 19 rather than eight electrodes. Rubin *et al.* [60] had two epileptologists label cases as seizure or no seizure. Compared with ground truth data, the epileptologists achieved a combined 70% sensitivity and 96% specificity for seizure detection. The reduced arrays were believed to contribute to the inferior sensitivity score. In a similar study, Herta *et al.* compared human expert seizure annotations of intensive care unit (ICU) EEG recordings with an automated electrode reduction model [61]. A step-wise method reduced the electrodes, calculating the sensitivity and specificity for each eliminated electrode. A minimum of nine electrodes was deemed necessary to detect the same ictal patterns as the human experts.

Indeed, since much data is discarded during pre-processing steps, beginning with the maximum amount of data may be advantageous. Further, a complete electrode set-up enables the EEG recording to capture the seizure activity's complete spatial and temporal evolution compared with the sparse spatial coverage by a few electrodes. This substantially helps to differentiate between seizure activity, ‘normal’ EEG and artefacts, as artefacts often contaminate EEG due to muscle activity or movements. A higher number of electrodes used in monitoring provides a higher chance that some of the electrodes capture mostly brain activity (rather than artefacts) which can be clearly assigned to a seizure.

For instance, if an individual experiences an unexpected seizure type not captured in their ward stay (i.e. a focal to bilateral tonic-clonic seizure occurring in someone diagnosed with focal seizures), a channel or electrode in only one or two regions may miss valuable information. Additionally, though channel selection studies highlight the wealth of possibilities in channel selection, the model combinations and interchangeably used terms (electrode and channel) make delineation, comparison and reproducibility of best-in-class models challenging. These impositions illustrate the need for further research.

## 7. Benefits of reduced electrodes/channels

Achieving the balance between data quality and fewer electrodes in a portable EEG system would greatly evolve home EEG monitoring. In particular, a low channel device could provide reliable information about seizure frequency to monitor treatment and get a better ground truth for drug trials, thus improving patient comfort while maintaining diagnostic reliability.

Accuracy of patient-reported seizures is highly dependent on seizure type, and patient ability [62]. Such dependence can lead to under-reporting of seizures, a long-standing, significant clinical problem [62–70], which patients themselves are aware of [71]. Ultimately, inaccurate seizure reporting impacts patient diagnosis and treatment, and conflates the evaluation of medication efficacy [72].

Non-invasive mobile EEG systems offer solutions to such challenges and have been reported by patients as valuable in seizure detection and management [73]. Reducing the number of electrodes required in these systems or incorporating channel selection methods would extend their adoption in the following scenarios. In the first scenario, channel selection may be a second step for first-seizure or new-diagnosis patients after data is collected in the ward using the full set of electrodes. A low-channel device could be worn beyond the clinic if seizures were not recorded during the ward stay. This would alleviate the need for patients to manually log seizures, possibly yielding more reliable information about seizure occurrence and frequency, and providing a ground truth for comparisons during medication trials.

In the second scenario, the channel selection methods can augment other neuroimaging data, such as MRI, to corroborate imaging findings and aid the determination of treatment pathways. In the third scenario, channel selection methods would be valuable for highlighting the optimal brain regions for placement of invasive or ambulatory devices for ultra-long-term monitoring. In such cases, the onset zone is likely to be established using gold standard clinician assessment.

In the clinical setting of the ICU, the use of a reduced number of electrodes for continuous EEG (cEEG) without compromising seizure detection accuracy is a common practice [74–76]. The nature of critical care provided in neonatal and adult ICUs makes it challenging for technicians to apply and review a full electrode set, resulting in the current consensus on the reliability and use of a reduced number of electrodes [77,78].

Several studies in paediatric and adult ICUs demonstrate that electrode reduction does not significantly compromise the sensitivity and specificity of seizure detection. One recent study exploring the impact of electrode reduction in a paediatric epilepsy monitoring unit showed the sensitivity and specificity of human detection were only marginally lower for the reduced montage (0.65) compared with the full montage (0.76) [79]. Another study evaluated simultaneous recordings from a 23-electrode EEG and a six-electrode EEG in 12 adult ICU patients to detect non-convulsive seizures, with two neurophysiologists achieving consensus regarding the seizure activity in all 12 patients [80]. Thus the importance of fewer electrodes has been emphasized [61], especially in neonatal ICUs [81]. However, a recent systematic review of amplitude-integrated EEG (aEEG), which uses just two to four electrodes to diagnose neonatal seizures, highlighted the relatively low sensitivity and specificity and called for future studies to address this concern [82].

All the studies noted that including a human assessor could greatly enhance automated seizure detection; thus, electrode reduction may be less problematic with human assessors involved. Perhaps it is only in the non-human evaluation of EEG recordings, such as in computer-based automated seizure detection, where electrode reduction poses a problem. Therefore opportunities to boost reduced electrode EEG recordings and channel selection methods may be through incorporating data from diffusion magnetic resonance imaging (dMRI) and wearables.

### 7.1. Combining electroencephalography with diffusion magnetic resonance imaging data

Advances in neuroimaging of brain structural connectivity may offer a unique solution whereby a patient's MRI scan guides electrode placement. The role of structural connectivity in focal seizures, as observed in the brain's white matter, is largely established [83]. Therefore imaging such as dMRI presents a worthwhile aid for patient-specific determination of electrode placement. Diffusion MRI enables the assessment of white matter fibre or tissue characteristics [84] and the derivation of metrics that represent the microstructural organization [85]. Further explanation of dMRI is beyond the scope of this review. However, the potential for dMRI to augment EEG in a range of diseased populations has recently been shown [86]. In epilepsy populations specifically, a relationship was identified between source localization metrics and alterations in white matter tracts [87–90] and event-related potentials and diffusion tensor metrics [91]. Further, microstructural white matter alterations have been shown to be predictive of surgical outcomes using dMRI [92].

**Figure 3.**
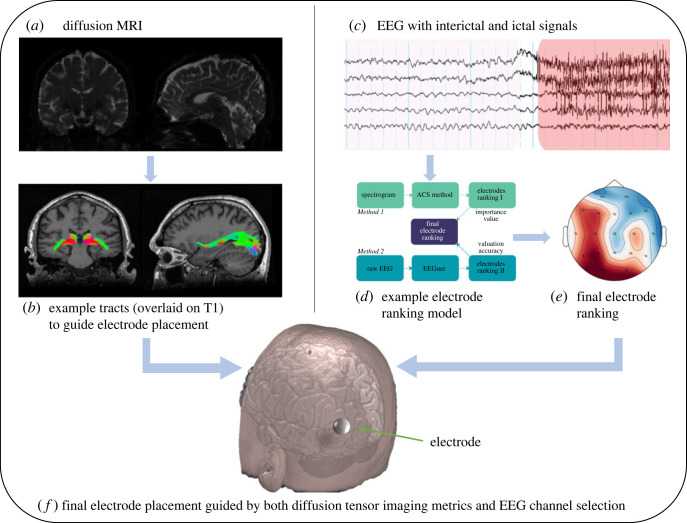
A proposed method for dMRI-guided EEG electrode placement. The MRI scans (T1 and diffusion) (*a*) are processed through an imaging pipeline to obtain tractography and tract-weighted tensor metrics (*b*). The EEG signals (*c*) are processed, applying machine learning (ML) and automated channel selection (ACS) for seizure detection (*d*), and the critical electrodes specific to that patient are selected and ranked (*e*). Further, clinicians could use the results of the tract-weighted tensor metrics and the patent-specific EEG electrodes to guide optimal placement for a wearable or implantable device.

Although the previous works focused on concurrently recorded dMRI and EEG using the standard full set of electrodes, the advantages of including dMRI in an analysis pipeline are evident. Individualized dMRI data combined with the automated ranking of EEG signals can support a patient-specific pipeline for seizure onset zone selection by indicating the optimal electrode placement and the number of electrodes required to optimize automated seizure detection. Figure 3 portrays a theoretical process for using EEG and dMRI to obtain the optimal electrode placement. Items (*a*) and (*b*) were imaging pipelines explored in our previous work [93] while (*c*) and (*d*) illustrate a schematic workflow for the addition of EEG data, resulting in (e), identifying the optimal implant site for a long-term sub-scalp electrode or device.

However, obtaining a dMRI scan is costly, and its analysis with EEG can add additional time and computational expense. Realistically, it would take several hours for the dMRI metrics to be produced, making them unrealistic for real-time monitoring. An alternative would be to use interictal spikes from a short (less than 20 min) recording to guide automated channel selection; however, this requires interictal spikes to be present in the recording and also align with clinical information.

To realize the vision of combining multiple data modalities in real time, hospitals must be equipped with high-throughput computer systems and hardware, an unlikely position for most publicly funded hospitals, especially those in low-resource settings. Therefore the combination of dMRI and EEG, though valuable, remains feasible primarily for the pre-monitoring stage where clinicians might use dMRI metrics to guide the placement of EEG electrodes before monitoring or electrode reduction in home monitoring. Fusing EEG with data from wearables may be another practical solution.

### 7.2. Combining electroencephalography with wearable data

Non-invasive wearable devices can also augment data recorded from fewer EEG channels. A recent systematic review and meta-analysis evaluated the utility of non-invasive wearable devices in identifying epileptic seizures [94]. The review highlighted that biometric physiological data (i.e. three-dimensional accelerometry, surface electromyography, heart rate and heart rate variability) were beneficial in detecting tonic-clonic and psychogenic non-epileptic seizures with reasonable sensitivity and varied false alarm rates.

Electrocardiogram (ECG) data has been commonly evaluated as an added seizure marker, with some studies finding it improved seizure detection when combined with a limited channel EEG [95]. Other biomarkers of seizure severity gleaned from non-brain wearables include autonomic changes, frequency, type, post-ictal position and seizure duration [96,97].

Given the promise of these devices and EEG channel selection methods, researchers should combine the two modalities to determine their suitability for augmenting the EEG data so that seizures can be accurately and reliably detected. Research shows patients with epilepsy prefer wearables with higher sensitivity over those with lower false alarm rates [98]. Therefore, combining a reduced-channel EEG with a wearable may be an appealing option for improving the patient experience during the diagnosis and monitoring phases.

## 8. Future of scalp electroencephalography wearables

Advances in EEG wearables primarily seek to reduce the number of electrodes and fuse innovative techniques with the optimal locations for securing the electrodes to the scalp. An extensive review of modern EEG wearables with reduced electrodes is available elsewhere [99]. However, noting some of the clinical and research-grade devices and their benefits here is worthwhile.

The ‘Insight’ device by EMOTIV (EMOTIV) offers five channels, while the Neurotrail by Neuro-Pro AG starts at eight electrodes and can be reduced to one; both utilize dry electrodes. The Neurotrail exemplifies the multi-modal physiological signal fusion discussed in §7.2, as it contains a gyroscope and accelerometer to support its algorithms in real-time artefact detection and removal. The Advanced Brain Monitoring device (Advanced Brain Monitoring Inc.) also offers multi-modal signal fusion, using three frontopolar channels to record EEG, electrooculography and electromyography signals. Ceribell Inc. offers an eight-channel seizure monitoring device with an inbuilt seizure alert system, making it advantageous for low-resource or intensive care settings where experienced clinicians may not be available.

The REMI device (Epitel) attaches to the skin using a disposable sticker containing a hydrogel that acts as both the adhesive and the conductive gel [100]. The attachment method enables continuous 24-hour capture of EEG for up to a week; the recording is then given to clinicians for data analysis. Though Epitel's device is comparable to a full-electrode EEG recording, a key shortcoming is the hydrogel base, which dries over time, resulting in similar outcomes to the conductive gel electrodes [99]. However, the requirement to place it on a hairless skin patch (such as behind the ear) may be an advantage of the device given the growing interest in ‘behind-the-ear’ or ‘in-ear’ EEG.

Indeed, aside from the REMI device, most of these devices are hardly discreet, deterring their use beyond a clinical or research setting. Behind-the-ear or in-ear EEG addresses such concerns through discreet placement, and recent studies have shown it can achieve a reasonable level of accuracy in seizure detection [101,102].

At the forefront of the EEG wearable innovation are liquid metal-based wearables [103]. Gallium-based liquid metals are a promising class of materials, with a key advantage being the concurrent offering of low toxicity and high electrical conductivity while maintaining malleability [104,105]. The products resulting from liquid metals have been termed ‘soft electronics’, as their properties afford moulding into a range of desirable formations that are micrometres thin, stretchable, discreet and attachable using stickers [103]. Still, the inherent challenges to be addressed before liquid metal products become commonplace include bio-compatibility, toxicity, solidification, oxidation and corrosion, which researchers are beginning to investigate [106,107].

## 9. Discussion and conclusion

Since EEG is an established and reliable method for capturing and identifying seizure activity, evolving beyond the current scalp electrode requirements would amplify its utility. Given the challenges in automated seizure detection and logging, a portable, reduced electrode device presents an invaluable solution to improve diagnosis and the patient journey. A portable device with only a few electrodes would enable long-term recordings, providing rich neurophysiological data for patient-specific data processing.

The analysis of existing works highlights the inspiration researchers can take from settings like the ICU to improve electrode reduction study designs and methodologies. In epilepsy, automated seizure detection models could be refined and enhanced by adding selective neuroimaging biomarkers and metrics from dMRI. The opportunity to improve the biometric and physiological data capture while maintaining reliable seizure assessment may lie in the augmentation of EEG with additional data from non-invasive wearables.

A resurgence of research into electrode reduction in the clinical setting will support the development of portable, reliable devices, thereby enabling long-term monitoring and enhancing patient quality of life. Future research should prospectively explore precisely how much long-term data with a full electrode set is required to produce an optimal channel selection outcome. Similarly, researchers should look to quantify the number of reduced electrodes, that are sufficient to produce a diagnosis or act as an effective long-term monitoring device when combined with wearable device data. Personalized models for such multi-modal data analysis would also be beneficial.

Transforming the existing solutions from a diagnosis-only state to a seizure logging and management aid for clinicians and patients will enhance the patient journey. Neurobehavioural and psychiatric comorbidities of epilepsy might be better understood [108,109]. The diagnosis and treatment pathways for epilepsy comorbidities could be optimized [110,111]. Overall, a faster, reliable diagnosis, with fewer hospital visits, would improve the patient journey in low-, middle- and high-income countries, offering a safer and greater quality of life.

## Data Availability

This article has no additional data.
